# Protease-Assisted Mild Extraction of Soluble Fibre and Protein from Fruit By-Products: A Biorefinery Perspective

**DOI:** 10.3390/foods12010148

**Published:** 2022-12-28

**Authors:** Andrea Fuso, Pio Viscusi, Susanna Larocca, Francesco Saverio Sangari, Veronica Lolli, Augusta Caligiani

**Affiliations:** 1 Food and Drug Department, University of Parma, Parco Area delle Scienze 27/A, 43124 Parma, Italy; 2 Sogis Industria Chimica Spa, Via Giuseppina 132, 26048 Sospiro, Italy

**Keywords:** protease-assisted extraction, fruit by-products, seeds, kernels, soluble fibre, protein

## Abstract

By-products from the fruit supply chain, especially seeds/kernels, have shown great potential to be valorised, due to their high content of macronutrients, such as lipids, protein, and fibre. A mild enzymatic assisted extraction (EAE) involving the use of a protease was tested to evaluate the feasibility of a cascade approach to fractionate the main fruit by-products components. Protease from *Bacillus licheniformis* (the enzyme used in the AOAC 991.43 official method for dietary fibre quantification) was used, and besides protein, the conditions of hydrolysis (60 °C, neutral pH, overnight) allowed us to dissolve a portion of soluble fibres, which was then separated from the solubilized peptide fraction through ethanol precipitation. Good protein extraction yields, in the range 35–93%, were obtained. The soluble fibre extraction yield ranged from 1.6% to 71% depending on the by-product, suggesting its applicability only for certain substrates, and it was found to be negatively correlated with the molecular weight of the fibre. The monosaccharide composition of the soluble fibres extracted was also diverse. Galacturonic acid was present in a low amount, indicating that pectin was not efficiently extracted. However, a predominance of arabinose and galactose monomers was detected in many fractions, indicating the isolation of a fruit soluble fibre portion with potential similarity with arabinogalactans and gum arabic, opening up perspectives for technological applications. The residual solid pellet obtained after protease assisted extraction was found to be an excellent fibre-rich substrate, suitable for being subjected to more “hard” processing (e.g., sequential pectin and hemicellulose extraction) with the objective to derive other fractions with potential great added economic value.

## 1. Introduction

The global food waste generation is estimated at 1.3 billion tons/year [1]. Fruit processing waste (e.g., peels, pods, seeds, skins, etc.), accounting for 45% of the total fresh weight, generated around 3.3 billion tons of carbon dioxide each year, due to landfill disposal or incinerators [2,3]. This unsustainable fruit waste management also results in a loss of valuable biomass and nutrients [4]. Indeed, many compositional studies suggested the potential utilization of fruit by-products as substrate for biorefinery in the production of high-value compounds [5]. Fruit seeds and kernels are especially rich in macronutrients (lipids, proteins, and dietary fibres) and secondary metabolites (e.g., antioxidants, phytosterols) [6], in some cases in concentrations higher than the edible parts of the fruit [7,8]. However, the presence of toxic substances in some kernels, for example the cyanogenic compounds in *Prunus* genus, have hindered the re-use of the whole kernels in the food industry [9]. 

Dietary fibres are certainly one of the most promising compounds to be extracted from fruit by-products because of their high quantity [10] and their beneficial effects on health. Fibre emerged as the leading product segment in the Europe bioactive ingredients market and accounted for over 20% of the total industry in 2015 [11]. Beside their fundamental role as technological additives, fibres are also recognized as an important part of a healthy diet (by preventing cardiovascular diseases, obesity and diabetes). The global market for high fibre content foods has been estimated to be continuously growing, driven by the consumer focus on health, wellbeing and by the increasing awareness on the benefits of fibre-rich diets [12]. 

Despite eating more fibres being currently considered mandatory for a healthy diet, the use of fibre-rich integral plant foods, however, poses technological and organoleptic problems related to food acceptability, often requiring fibre extraction and modification. In this sense, there is a growing need to develop more sustainable and innovative technologies of extraction and fractionation. Among recent promising environmentally friendly methods, enzyme-assisted extractions (EAE) allow us to disrupt the structural integrity of the plant cell wall [3], thus enhancing the extraction of fibres and other nutrients, minimizing the use of solvents and heat, and preserving the structure and functional properties of the extracted biomolecules. 

However, the extraction of a single class of biomolecules from food by-products is currently considered to be a limiting approach, due to the still high amount of residual biomass left un-valorized. In contrast, the application of cascade biorefinery processes to recover the most of valuable compounds could meet the important challenge of giving added value to the whole fruit processing value chain, justifying the additional investment related to the new technologies. Despite this, development of fruit waste biorefineries is limited, mainly due to the lack of information on feedstock availability, composition, process design, and scale-up. Hence, adapting the biorefinery strategy with integrated approaches based on green (enzyme-assisted) methods would lead to products with significantly higher values compared to the current applications of the fruit residual biomass. In support of this, combining the mild extraction of soluble fibres and protein could be an interesting approach to tackle the current need to shift food products and healthy diets towards higher intakes of both plant protein and fibres. As a matter of fact, there is a growing number of food sectors requiring plant-based protein (e.g., vegan product market, and meat substitution) and also new dietary fibre (e.g., functional foods, nutraceuticals, and prebiotics). All these sectors may benefit from a new and wider portfolio of available ingredients.

In this context, this work is focused on the valorisation through biorefinery approach of underexploited fruit biomass streams, namely seeds, peels and kernels. Fruit by-products samples were chosen to cover different botanical families, but also on the basis of the morphological characteristics that may have an influence on the extractive processes (e.g., seed size, hardness and thickness of the lignocellulose shells). The general aim was to investigate the molecular composition of fruit by-products and to test mild EAE by using protease for the cascade recovery of nutrients. In particular, this study deals with the purification and chemical characterization of soluble dietary fibres simultaneously extracted with protein through EAE with protease and with the investigation of the residual biomass composition obtained after this process in a total biorefinery perspective.

## 2. Materials and Methods

### 2.1. Reagents

D-fructose, D-glucose, D-arabinose, D-rhamnose, D-ribose, D-xylose, D-fucose, D-galactose, D-mannose, D-glucuronic acid, D-galacturonic acid, phenyl β-D-glucopyranoside, dimethylformamide (DMF), trifluoroacetic acid (TFA), ammonium hydroxyde and N,O-Bis(trimethylsilyl)trifluoroacetamide (BSTFA) were purchased from Sigma-Aldrich (Taufkirchen, Germany); ethanol was purchased from Carlo Erba (Milan, Italy); bidistilled water was obtained using Milli-Q System (Millipore, Bedford, MA, USA), while methanol from VWR International (Milan, Italy).

### 2.2. Fruit By-Products Collection and Characterization 

The analyses were carried out on fruit-derived by-products samples. These were either purchased or supplied by fruit and vegetable processing companies. In detail, samples comprised different fruit by-product covering different species and characteristics: stone fruits of the *Prunus* genus as cherry kernel (*Prunus avium*) and mango seeds (*Mangifera indica* L.), citrus fruits as orange peel and seed (*Citrus × sinensis*), lemon seeds and lemon peels (*Citrus limon*), and finally a less common specie of the Rosaceae family, namely loquat kernel (*Eriobtria japonica*).

Samples were treated with liquid nitrogen and milled through laboratory blenders and then characterized in terms of proximate composition according to official methods of analysis [13]. Moisture was determined in an oven at 105 °C for 24 h. Total ash was determined through mineralization at 550 °C for 5 h. Proteins were determined with a Kjeldahl system (DKL heating digestor and UDK 139 semiautomatic distillation unit, VELP SCIENTIFICA) by using 6.25 as the nitrogen-to-protein conversion factor. Total and soluble fibre content were determined by the AOAC 991.43 official enzymatic-gravimetric method for dietary fibres [14].

### 2.3. Protease Assisted Extraction

**Figure 1:**
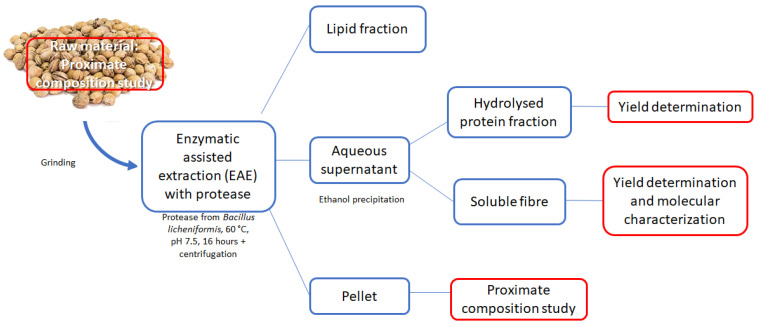
Representation of the fruit by-products one-step fractionation process.

Twenty grams of each sample underwent enzymatic assisted extraction with the employment of an alcalase of microbial origin, namely protease from *Bacillus licheniformis* (EC 3.4.21.62). The EAE was performed for each fruit by-product at the optimal conditions of temperature and pH for the enzyme (60 °C and pH 7.5, respectively). An enzyme/substrate ratio of 1:100 (*w*/*w*) was mixed with a phosphate buffer solution (10 mM Na_2_HPO_4_/NaH_2_PO_4_) and hydrolysed overnight (16 h), then heated at 90 °C for 10 min to inactivate the enzyme. The hydrolysed substrate was centrifuged at 3900 rpm at 4 °C for 40 min to separate the aqueous supernatant from the insoluble residue (pellet) and eventually from a portion of lipid fraction on the surface. The top surfaced oil (when present) was directly recovered. The solid residue (pellet) was recovered and characterized as described in Section 2.5. Ethanol (95% *v*/*v*) was then added to the supernatant in a 4:1 ratio in order to obtain the precipitation of soluble fibre that had been simultaneously extracted from the matrices along with proteins/peptides. Proteins were then purified from the precipitated soluble fibres by centrifugation (3900 rpm, 4 °C, 30 min). Then, the supernatant was recovered to determine total nitrogen and the pellet constituted by the alcohol insoluble residue was recovered, washed again with ethanol and characterized according to paragraph 2.4. The process workflow is represented in Figure 1.

### 2.4. Characterization of Soluble Fibre 

#### 2.4.1. Residual Ash and Protein

The soluble fibres extracted were characterized in terms of purity by quantifying the total protein and ash content. Official methods were used according to Section 2.2.

#### 2.4.2. Molecular Weight

The molecular weight of soluble fibres was also evaluated, in order to understand their potential use in the food supply chain, through High-Performance Size-Exclusion Chromatography coupled with Refractive Index Detector (HPSEC-RID). The samples were dissolved in ultrapure water at a concentration of 10,000 ppm, then centrifuged at 7000 rpm for 20 min at 4 °C and finally filtered through a 0.45 µm nylon membrane. An Agilent 1260 Infinity II LC system equipped with a refractive index detector (RID) (Agilent, Santa Clara, CA, USA) was used. Ultrapure water was also used as eluent, at a flow rate equal to 0.7 mL/min, and a PL aquagel-OH mixed-M column, 7.5 mm × 300 mm, 8 µm was employed for the separation (Agilent, Santa Clara, CA, USA). The injection sample volume was set at 10 µL, column temperature 30 °C and RID temperature 35 °C. Standard pullulans having known molecular weight ranging from 6000 to 200,000 Da were used for the calibration curve. 

#### 2.4.3. Monosaccharide Composition

The total quantity of soluble fibres obtained after precipitation through ethanol addition to the supernatant, as well as their monosaccharide composition, was investigated for every sample. The analysis was performed following a method proposed in literature based on acid hydrolysis followed by GC-MS injection [15].

### 2.5. Proximate Composition of Residual Pellet after EAE

A residual pellet was obtained on the bottom of the tube after EAE (Figure 1). This pellet was characterized in terms of proximate composition, namely dry weight, residual proteins, lipids, ash, and total dietary fibre. Official methods were used to quantify all the components, as already reported in Section 2.2.

### 2.6. Determination of Extractions Yields 

The total yield of soluble fibre was determined as percentage (%), calculated by dividing the sum of monosaccharides determined in Section 2.4.3 by the absolute amount of soluble fibre of the starting material determined by the AOAC gravimetric method.

The enzymatic extraction yield (%) of proteins was calculated by comparing the total nitrogen after the enzymatic hydrolysis in the supernatant and the total nitrogen determined before the proteolysis in the raw materials. 

## 3. Results and Discussion

### 3.1. Proximate Composition of the Raw Materials (Fruit By-Products)

The fruit by-products compositional analysis is of great importance for their further exploitation. As reported in Table 1, the proximate composition (ash, protein, lipids, soluble, insoluble, and total fibres) of the different raw materials (seeds, kernels, and peels) considered in this study revealed different nutritional contents based on the fruit type/category. 

The protein fraction accounted for about 4–15% on dry matter basis, and especially lemon seeds resulted as a good source of protein (15.3 ± 0.2%), whereas cherry kernels had the lowest content (4.5 ± 0.2%). Fruit seeds/kernels mainly turned out to be good sources of dietary fibre, as previously documented [10]. Lemon seeds and cherry kernels were found to be the richest ones in total dietary fibre content (with percentages on dry matter equal to 76% and 81%, respectively) even though they did not have high amounts of soluble fibres (4% and 6%, respectively). On the contrary, citrus peels contained very high amounts of soluble fibre (around 20%), likely due to the contribution of pectin fraction. Compared to these percentages, total fibre was low in mango seeds and loquat kernels, but the latter had a well-balance proportion between soluble and insoluble fractions. 

Digestible carbohydrates (of which percentage values were obtained by difference and indicated as “others”) represented the major nutrients in mango seeds and loquat kernels, thus being potential energy food sources. However, the potential presence of starch could interfere with soluble fibre purification after protease assisted extraction. In mango seeds, the presence of starch has been confirmed by Patiño-Rodríguez and colleagues, who determined a concentration close to 30% within the same by-products [16]. No studies were found in the literature regarding the presence of starch in the other seeds and kernels considered in this work.

### 3.2. Yield Determination for Soluble Fibre and Protein after Protease Assisted Extraction

As already mentioned, one of the main purposes of this work was to simultaneously recover both fruit protein and soluble fibre. After protease reaction and ethanol addition, the fruit proteins were recovered in the form of protein hydrolysates in the hydro-alcoholic supernatant, whereas the fibre fraction was a precipitate. 

Table 2 shows the extraction yields, expressed as a percentage of protein and soluble fibres in the six samples considered. Regarding the protein fraction, yields were on average high (60%), suggesting good activity of the protease employed on recalcitrant residues such as lignocellulosic seeds/kernels. The maximum values were obtained for citrus fruit peels (80% and 93% for orange and lemon peels, respectively), whereas the minimum yield (35 ± 2%) was determined for loquat kernels. These yields are on average very satisfactory, considering that similar results were previously obtained with less environmentally friendly methods (such as alkaline solubilisation/isoelectric precipitation extraction) and on similar matrices [17,18]. Moreover, as previously mentioned, this protocol also allows us to recover a fraction of dietary fibre (Table 2).

The total amount of soluble fibre in the ethanol precipitates was determined by the sum of the single monosaccharides freed up following acid hydrolysis (see Section 2.4.3). It was found that the protease assisted extraction had excellent extraction yields not only for protein but also for soluble fibres in some samples, namely cherry kernels (71%), mango seeds (33%) and orange peels (30%). On the contrary, in other samples significantly lower extraction yields were obtained, especially in the case of loquat kernels (1.7%), suggesting different characteristics of the soluble fibres or likely their different interactions and bond with the lignocellulosic structures. De Albuquerque and colleagues performed a similar experiment, evaluating the effectiveness of “mild” extractions (90–95 °C, atmospheric pressure and pH around neutrality) in various tropical fruits by-products. The authors quantified the soluble fibre content in the extract, and also in that case the quantity of fibres extracted from mango by-products was significantly greater than in other samples examined, followed by orange, while for passion fruit and acerola these quantities were found to be very low [19]. The extraction yields obtained by De Albuquerque’s group are on average higher than the ones in the present work, but this is not surprising since different extraction temperatures were used (90 °C against 60 °C) and the work was focused only on soluble fibre, while the primary objective of our process was the simultaneous extraction of proteins and fibres. An interesting fibre recovery equal to 55% was instead recently obtained on mango peels, employing α-amylase, protease and amyloglucosidase in sequence [20]. However, in our case this method would probably lead to an enhancement of fibre recovery, but to a decrease of protein purity due to the solubilization of starch-derived glucose within the hydroalcoholic solution. 

### 3.3. Molecular Weight of Soluble Fibre

The molecular weight of soluble fibre extracted from fruit by-products through EAE was investigated by High-Performance Size-Exclusion Chromatography coupled with a Refractive Index Detector (HPSEC-RID). The results are shown in Table 3.

From Table 3, it can be seen that all the samples were composed mainly of low molecular weight polysaccharides, lower than 6 kDa, or high molecular weight polysaccharides, higher than 200 kDa. Mango and orange by-products turned out to consist mainly of low molecular weight molecules, which could explain the decent extraction yield obtained for these matrices. In contrast, loquat and cherry kernels and lemon peels and seeds turned out to be composed mainly of high molecular weight polymers. 

Fractions with intermediate molecular weights were only detected in the samples derived from lemon peels and seeds. The soluble fibre extracted from lemon seeds turned out to be composed for 19% of polymers with a molecular weight in the range of 95 to 100 KDa, while the extract obtained from the peels was made of polysaccharides with a molecular weight ranging from 15 to 20 kDa for 8% of the total area. 

It is well known that the molecular weight of polysaccharides has consequences for their physicochemical, physiological and biological properties. In particular, molecular weight is strictly related to viscosity, which increases as the chain length. Therefore, biological and physiological activities positively associated with viscosity, such as antimicrobial and cholesterol-lowering activity [21], improve in a directly proportional manner with chain length. In addition, it has been proposed that the antioxidant property might be related to the molecular weight of polymers, being possibly influenced by the number of hydroxyl and hemiacetal groups of the polymer [21]. Molecular weight also affects the technological properties of carbohydrates: for example, β-glucans of different sizes affected breadmaking ability, increasing water binding capacity, and modified the texture and the ability to stabilize emulsions [22]. The presence of molecules with different molecular weights could be the starting point of possible future strategies for the preparative separation of polymers based on this feature, in order to be able to exploit them according to their functional properties.

### 3.4. Monosaccharide Composition

**Figure 2:**
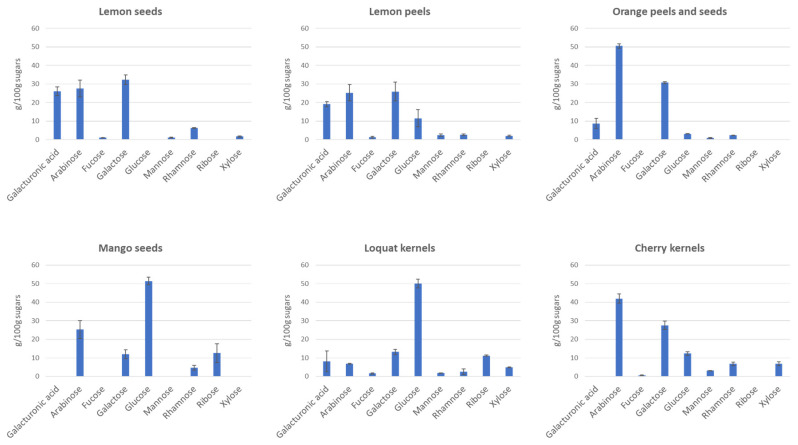
Sugar composition (expressed as g sugar/100 g total sugars) of six fruit by-products.

The characterization of soluble fibre was also carried out in terms of their monosaccharide profile by GC-MS. While this technique does not provide a full understanding of the chemical structure of the molecule, it gives important information about which monomers make up the polysaccharide, both in qualitative and quantitative terms. Histograms in Figure 2 show this composition for the six fruit by-products considered in this study. The determination of monosaccharide composition for each sample was performed in duplicate, and the results are reported as mean ± standard deviation.

Soluble fibre of loquat kernels and mango seeds were the only ones containing glucose as the main monosaccharides, suggesting a potential significant presence of starch, as highlighted in Section 3.1, which needs to be further evaluated in terms of the purification process. Ethanol, in fact, may cause the co-precipitation of starch with fibres [23]. In such case, the use of amylolytic enzymes, resembling conditions used for gravimetric quantification of soluble fibres [14] could be therefore appropriate, but it was excluded in this protocol to avoid the presence of simple sugars in the hydrolyzed protein fraction. Amylase and amyloglucosidase could be eventually used, if needed, directly on the soluble fibre/starch extract.

In Citrus fruit samples, namely orange by-products and lemon peels and seeds, the most present monosaccharides were arabinose (25–50%) and galactose (25–35%), followed by galacturonic acid (10–25%). A higher concentration of uronic acids was expected. The latter, in fact, accounts for about 65% of the monosaccharides present in citrus peels [24], as it is the main constituent of pectin, a polysaccharide known to be present in a high concentration in these by-products. However, it is important to underline that the methods generally used for pectin extraction involve diverse temperature and pH conditions, namely 80 °C and pH 2 [25], quite far from those used during the EAE used in our work. Thus, it is evident that the “mild” extraction methodology tested here is not totally suitable for pectin extraction. However, the protease assisted extraction allowed us to isolate, from citrus fruits by-products, a specific fraction of soluble fibre that is rich in arabinose and galactose, with possible specific applications.

More in general, arabinose and galactose were the two main monomers in four samples out of six. Their sum accounted for 82% of total sugars in orange by-products, for 60% in lemon seeds, for 51% in lemon peels and for 69% in cherry kernels, suggesting the presence of arabinogalactans as the main soluble fibre extracted in the conditions used. It has been indeed reported that arabinogalactans, unlike pectin, have better extraction yields at low temperatures and neutral pH, as obtained by Hamed and colleagues [26]. According to the literature, the presence of arabinogalactans has been shown in the pulp of peach [27], in pistachio shells [26], and in general in different fruit parts, such as apples [28], prickly pear peels [29], and carambola (starfruit) [30]. Arabinogalactans are polysaccharides having a molecular weight of about 58 kDa [31], composed mainly of galactose and arabinose and sometimes with lower amount of rhamnose and glucose units [26]. The presence of higher molecular weight molecules within all the analysed samples (Table 3) could be associated with the presence of polymers similar to gum arabic, which have been reported to have molecular weights in the range of 312–950 kDa and arabinose and galactose as prevalent monosaccharides [32]. In plants, arabinogalactans are often bound to proteins and represent the major proteoglycans within plant cell walls, and gum arabic is indeed made of arabinogalactans (AG) and arabinogalactan-protein (AGP) complexes [33]. The proximate composition of the soluble fruit fibre fractions extracted in this work was also investigated and turned out, on average and respect to the dry matter, to be made of 50% fibres, 30% proteins and 20% ash, indeed demonstrating a significant amount of protein in the soluble fibre fraction as for gum arabic. However, the percentage of residual protein is quite high, and it cannot be exclusively attributed to glycoproteins, also suggesting a small percentage of non-hydrolysed proteins that co-precipitates with fibres after ethanol addition. 

While a lot of unusual new sources of arabinogalactans, including plant seeds, have been recently found and summarized in a recent review [34], not many reports about their presence in fruit by-products are present, especially when fruit kernels are considered. This work lays the foundation for future studies, which could focus more on these by-products as a potential matrix for the recovery with good yields of this polysaccharide, which in recent years has also attracted attention for its immunostimulatory activity [35]. 

### 3.5. Proximate Composition of Residual Pellet 

**Figure 3:**
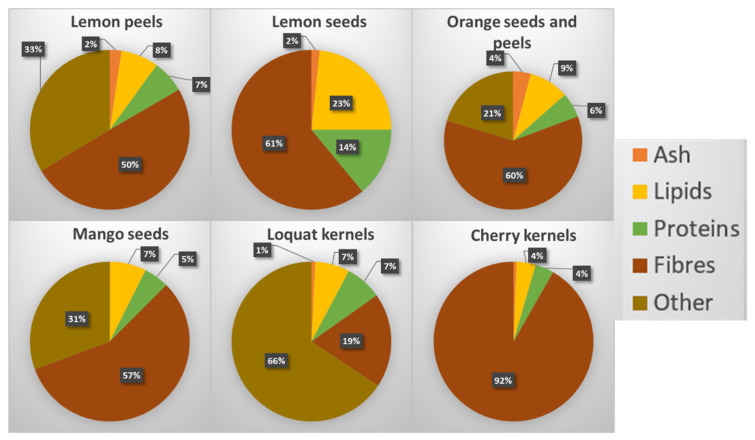
Proximate composition of the residual pellets remained after EAE. Each analysis was performed in duplicate and the results are expressed as their mean and as a percentage on dry matter (%DM). The percentage of “other” was calculated by the difference from 100.

After the employment of EAE, no separated lipid fraction was observed for any fruit by-products, while a significant amount of residual solid fraction (named as “pellet”) remained (Figure 1). In order to understand the possibility to further valorise it, from a perspective of circular economy and complete fractionation, a characterization of its components was performed in terms of dry matter, lipids, proteins, ash and total fibres. The pie charts below (Figure 3) report the results obtained. 

The results showed that all the residual pellets, with the exception of loquat kernels, were composed mainly of fibre. On average, five samples out of six presented a quantity of dietary fibre in the range 50–92%. These fibres are mainly insoluble (see Table 1), and since not the whole amount of soluble fibres was extracted during EAE (see Table 2), some residual soluble fibres are certainly also included in this portion. Since most of the samples examined are definable as lignocellulosic materials, it is assumed that most of this residual fibre consists of hemicellulose, cellulose and lignin as major fractions, which certainly cannot be extracted through a “mild” extraction like the EAE performed in the present work. These findings represent an interesting starting point for future works; in fact, through the use of “harder” extractions, such as extractions at higher temperatures and acid pH, it would be possible to recover pectin eventually present, and through autohydrolysis treatments the extraction of hemicelluloses could also be carried out. Therefore, it would be possible to further valorise these fruit and vegetable by-products, completing their fractionation and satisfying the circular economy concept. 

Regarding other compounds, the relative amount of protein present within the pellet still appears to be significant, especially for lemon seeds, and for this reason further studies are needed to understand whether the proteins are “free” and easily extractable in other media or complexed with other molecules. The lipid fraction remained almost totally within the pellets. This is not surprising since they are sometimes bound to the matrix, and for a total extraction from seeds they should be treated with organic solvent, in some cases also after acid treatment of the matrix, as for lipid extraction from raw cocoa beans [36].

## 4. Conclusions

This work fits in the context of agri-food by-products valorisation, from the perspective of trying to contribute to address issues regarding increased waste generation, environmental pollution and resource consumption. Some scraps from the fruit and vegetable supply chain, namely peels, seeds, and kernels, were subjected to enzymatic assisted extraction (EAE) with a protease aiming to simultaneously obtain proteins, lipids and fibres. 

As promising results, the enzymatic process based on the use of protease allowed us to: (i) Recover proteins such as protein hydrolysates; and (ii) preserve the structural integrity of the soluble fibre, which was recovered from the aqueous solution by ethanol precipitation, and the insoluble fibres, which mainly constituted the residual pellet.

Soluble fibres were recovered with high extraction yield, depending on the starting material. Most of the fibres were characterized by a dominant presence of arabinose and galactose moieties and a high molecular weight (>201 kDa), suggesting a chemical similarity of the obtained fibres with gum arabic. The presence of low molecular weight polymers (<6 kDa) was also revealed, especially in mango seeds and orange scraps. Since it is known how molecular weight affects certain technological and nutritional properties of polymers, a future approach based on the separation of these fractions might be considered. Finally, the study on the proximate composition of the residual pellets after EAE showed that it retained the total lipid fraction, the main portion was constituted by fibres, namely presumably cellulose, hemicellulose and lignin, but also the soluble fibres portion that was not extracted by the mild enzymatic treatment employed. These results suggest the possibility to further exploit the residual pellet for the extraction of lipids, and employing harder treatment, to extract other valuable fibre fraction (as hemicellulose and pectin), in order to fully valorise a very precious by-product that is today too much undervalued.

## Figures and Tables

**Table 1:** Proximate composition of fruit by-products as such, namely ground seeds, kernels and peels, expressed as percentage on dry matter (%DM). “Others” = obtained by difference, comprising digestible carbohydrates. Analyses have been performed in duplicate and the results are expressed as mean ± SD.

	Ash	Proteins	Lipids	Total Fibre (TDF)	Insoluble Fibre (IDF)	Soluble Fibre (SDF)	Others
Lemon peels	4.29 ± 0.23	7.26 ± 0.47	1.62 ± 0.09	58.42 ± 2.33	37.70 ± 1.23	20.72 ± 1.47	28.40 ± 1.82
Lemon seeds	2.45 ± 0.16	15.27 ± 1.09	6.37 ± 0.19	75.91 ± 3.03	75.43 ± 4.00	3.92 ± 0.23	-
Mango seeds	1.91 ± 0.06	5.11 ± 0.27	7.32 ± 0.52	37.07 ± 1.10	35.97 ± 1.06	1.11 ± 0.04	48.57 ± 2.57
Loquat kernels	2.74 ± 0.16	6.08 ± 0.36	1.12 ± 0.04	27.01 ± 1.08	14.89 ± 0.48	12.11 ± 0.86	63.04 ± 3.75
Cherry kernels	1.48 ± 0.09	4.54 ± 0.15	7.42 ± 0.22	80.90 ± 2.63	74.38 ± 2.20	6.54 ± 0.35	5.64 ± 0.22
Orange peels/seeds	5.56 ± 0.40	4.84 ± 0.34	1.91 ± 0.12	59.68 ± 1.94	26.65 ± 0.87	33.03 ± 1.07	28.01 ± 1.12

**Table 2:** Extraction yields, expressed in percentage, of soluble fibres (determined from total monosaccharide composition) and solubilized protein (determined by Kjeldahl method on the supernatant). Values are mean ± SD of replicates of independent extraction.

	Extraction Yield of Protein (% Respect to the Total Protein in the Raw Sample)	Extraction Yield of Soluble Fibres (% Respect to the Total Soluble Fibre in the Raw Sample)
Lemon peels	93 ± 7	6 ± 1
Lemon seeds	50 ± 2	9 ± 1
Mango seeds	42 ± 6	33 ± 2
Loquat kernels	35 ± 2	1.6 ± 0.3
Cherry kernels	70 ± 2	71 ± 5
Orange peels and seeds	80 ± 2	29.8 ± 0.2

**Table 3:** Molecular weight distribution (Peak Area %) of soluble fibre extracted by EAE from different fruit by-products.

	Molecular Weight (kDa)			
	<6	15–20	96–100	>200
	Peak Area (%)			
Lemon peels	2	8	-	90
Lemon seeds	5	-	19	76
Mango seeds	70	-	-	30
Loquat kernels	21	-	-	79
Cherry kernels	3	-	-	97
Orange peels and seeds	72	-	-	28

## Data Availability

Data is contained within the article.
